# Exploiting the circuit breaker cancer evolution model in human clear cell renal cell carcinoma

**DOI:** 10.15698/cst2020.08.227

**Published:** 2020-06-25

**Authors:** James J. Hsieh, Emily H. Cheng

**Affiliations:** 1 Molecular Oncology, Department of Medicine, Washington University, St. Louis, MO 63110, USA.; 2 Human Oncology and Pathogenesis Program, Memorial Sloan Kettering Cancer Center, New York, NY 10065, USA.; 3 Department of Pathology, Memorial Sloan Kettering Cancer Center, New York, NY 10065, USA.; 4 Department of Pathology and Laboratory Medicine, Weill Cornell Medical College, Cornell University, New York, NY 10065, USA.

**Keywords:** kidney cancer evolution, circuit breaker, VHL, HIF, PBRM1, MTOR

## Abstract

The incessant interactions between susceptible humans and their respective macro/microenvironments registered throughout their lifetime result in the ultimate manifestation of individual cancers. With the average lifespan exceeding 50 years of age in humans since the beginning of 20^th^ century, aging – the “time” factor – has played an ever-increasing role alongside host and environmental factors in cancer incidences. Cancer is a genetic/epigenetic disease due to gain-of-function mutations in cancer-causing genes (oncogene; OG) and/or loss-of-function mutations in tumor-suppressing genes (tumor suppressor genes; TSG). In addition to their integral relationship with cancer, a timely deployment of specific OG and/or TSG is in fact needed for higher organisms like human to cope with respective physiological and pathological conditions. Over the past decade, extensive human kidney cancer genomics have been performed and novel mouse models recapitulating human kidney cancer pathobiology have been generated. With new genomic, genetic, mechanistic, clinical and therapeutic insights accumulated from studying clear cell renal cell carcinoma (ccRCC)–the most common type of kidney cancer, we conceived a cancer evolution model built upon the OG-TSG signaling pair analogous to the electrical circuit breaker (CB) that permits necessary signaling output and at the same time prevent detrimental signaling overdrive. Hence, this viewpoint aims at providing a step-by-step mechanistic explanation/illustration concerning how inherent OG-TSG CBs intricately operate in concert for the organism's wellbeing; and how somatic mutations, the essential component for genetic adaptability, inadvertently triggers a sequential outage of specific sets of CBs that normally function to maintain and protect and individual tissue homeostasis.

## INTRODUCTION

On average a human adult body encompasses fifty trillion cells (~5x10^13^) with a daily turnover of hundred billion cells (~10^11^) within which each contains two copies of ~three billion base-pair DNA haploid genome. Approximately, the routine maintenance of a healthy human body necessitates ~10^21^ DNA base pairs transaction every day. Remarkably, despite this astronomical chemical challenge genome integrity is inherently maintained by sophisticated DNA replication, proof-reading, and repair mechanisms evolved over billion years. However, the precision of DNA transaction in multi-cellular complex organisms can only be near-perfect to allow finite replication errors needed for genetic adaptation and thereby evolution. Nevertheless, individual cells are equipped with intrinsic cell death machineries to assure continuous genome integrity, which works alongside with immune system's surveillance, killing, and removal of detected pre-cancer/cancer cells [1–3]. The human genome encodes ~20,000 protein coding sequences of which 2-10% are cancer-related genes such as oncogenes (OGs) and tumor suppressor genes (TSGs) [2, 4]. Notably, these cancer-related genes normally participate in key biological processes such as embryonic development and tissue homeostasis such as MLL (a Trx-G gene) and BMI-1 (a Pc-G gene)[5]. In response to imminent or chronic tissue stresses such as ischemia tissue injury these “specialty genes” are called upon to act appropriately till resolution of respective insults [2, 5–6]. Hence, we envisioned that specific oncogene and tumor suppressor gene pairs (OG-TSG) could function as regulatory modules similar to electrical circuit breakers (CBs) that mitigate power/signal overload [5]. Additionally, these OG-TSG CBs can interconnect to provide additional layers of regulation and safety that operate in a tissue context-dependent manner, which helps explain the tissue-type specific propensity of dysfunction in certain OG and TSG [7]. With these basic principles, hereafter we will begin to explore this hypothetic OG-TSG CB cancer evolution model inspired when we studied clear cell renal cell carcinoma (ccRCC) in human, mouse, and cell line models.

### Kidney cancer

Kidney cancer accounts for ~2% of all cancer diagnoses (~74,000; ~300,000) and deaths (~15,000; ~134,000) annually at the United States and the World, respectively[8, 9]. Renal cell carcinoma (RCC) encompasses a large heterogeneous group of cancers derived from renal tubular epithelial cells, which encompasses >10 molecular and histopathological subtypes [10]. RCC major subtypes (≥5%) include clear cell RCC (ccRCC) at ~75%, papillary RCC (pRCC) at ~15%, and chromophobe RCC (chRCC) at ~5% [11]. Accordingly, metastatic ccRCC (mccRCC) accounts for most of the kidney cancer mortality [12]. Although mccRCC is refractory to conventional chemotherapy, marked therapeutic advances have been made over the past 15 years, culminating in 15 FDA-approved agents for mccRCC [9]. These agents exploit seven molecular mechanisms: (**1**) IL-2 and (-Interferon are cytokines; (**2**) Sorafenib, Sunitinib, Pazopanib, Axitinib and Bevacizumab inhibit VEGF pathway; (**3**) Cabozantinib inhibits VEGFR2, cMET and AXL; (**4**) Lenvatinib inhibits VEGFR2 and FGFR; (**5**) Everolimus and Temsirolimus are inhibitors of mTORC1; (**6**) Nivolumab, Avelumab, and Pembrolizumab are PD-1/L1 blocking antibodies; and (**7**) Ipilimumab is an anti-CTLA-4 antibody [13]. However, mccRCC remains lethal and treatment response is highly heterogeneous among patients upon individual treatments [14], likely due to tumor genomic and host genetic heterogeneities.

### The genomics of ccRCC

Modern multi-omics of human ccRCC consisting of genomics, transcriptomics, proteomics and metabolomics not only highlights its profound inter-/intra-tumor heterogeneity but also showcases underline cancer evolution constraints that could be exploit as therapeutic biomarkers [14–25]. The most conspicuous genomic event in ccRCC is the near universal (>90%) one copy loss of the short arm of Chromosome 3 [26]. The most striking genomic discovery in ccRCC is the extreme chromosomal proximity of the four most prevalently mutated kidney cancer TSGs—*VHL* (~80%), *PBRM1* (29-46%), *SETD2* (8-30%), and *BAP1* (6-19%)—spanning chromosome 3p21-3p25 [6, 27–28]. Hence, one genetic event incurred the 3p loss in renal epithelial cells simultaneously creates a haplo-insufficient state of four critical ccRCC TSGs. Remarkably, 3p loss represents the first somatic driver event in sporadic ccRCC, which takes place during adolescence that predates the most common second somatic event by ~20 years, i.e., the genetic/epigenetic inaction of the *VHL* gene, giving rise to the most recent common ancestor (MRCA) within a given ccRCC patient whose ccRCC is eventually diagnosed at 60 years of age [29]. Notably, unlike most cancers that are initiated by gain-of-function mutations in OGs ccRCC results from sequential losses of TSGs [6].

### The shared genetic events of hereditary and sporadic in human ccRCC

The Von-Hippel Lindau (VHL) disease, an autosomal dominant hereditary cancer syndrome caused by the loss-of-function germline mutation in the *VHL* gene [30], is characterized by the development of hemangioblastoma of the central nervous system and retina, ccRCC, and pheochromocytoma [31–32]. It was later demonstrated that *VHL* is inactivated in ~90% of sporadic ccRCC through either genetic mutation or promoter methylation [9, 33]. Biallelic inactivation of the TSG *VHL* is therefore established as an early event in both germline mutant VHL-associated and sporadic ccRCC [6].

## DISCUSSION

### The VHL-HIF-hypoxia-metabolism

Studies on oxygen sensing led to the discovery of Hypoxia-Inducible Factors (HIFs) [34]. VHL is a multipurpose adaptor protein and chiefly effects as the substrate recognition module of the VCB (VHL-Elongin C-Elongin B)-Cul2 E3 ligase which ubiquitinates HIF-1□ and HIF-2□ [35]. Under normal oxygen conditions, HIF1/2□ is prolyl hydroxylated by EGLN, ubiquitinated by VCB-Cul2-VHL, and rapidly degraded by the 26S Proteasome [36]; whereas under low oxygen conditions such as high altitude or ischemia, HIF□ is stabilized to initiate a myriad of hypoxia-specific transcriptional programs [34, 36–38]. The pathologic loss of VHL in ccRCC results in persistently elevated HIFs accounting for the observed clear cell morphology and highly vascularity [11, 12, 38–40]. However, the long latency (>30 years) for VHL syndrome patients to develop ccRCC [31] and the insufficiency of VHL loss alone to induce ccRCC in mice [41] argue for the necessity of cooperative events [42].

### ccRCC signifies prevalent loss-of-function mutations in TSGs at the renal epithelium

Unlike many cancers that originate from gain-of-function mutations in OGs such as *EGFR* and *RAS*, ccRCC manifests with prevalent loss-of-function mutations in TSGs, making the development of predictive biomarkers for individual targeted therapies and/or immunotherapies extremely challenging. Nevertheless, new therapeutic modalities, novel genetically engineered mouse models, clinically relevant patient-derived cell line/xenograft models, and outcome-based biomarker studies altogether have begun to shed light on how these seemingly distinct research areas are in fact exquisitely interconnected [43]. Here, we wish to update essential findings in ccRCC, and present a novel concept of “Interconnected OG-TSG Circuit Breaker Cancer Evolution Model in ccRCC”.

### The first oncogenic driver event in ccRCC

In hereditary VHL-loss ccRCC, the first genetic event is the inheritance of a loss-of-function copy of the VHL gene; whereas in sporadic ccRCC, the first genetic event is the loss of chromosome 3p. The ensuing epi/genetic event in developing both kinds of ccRCC converges on the complete inactivation of VHL [6]. Hence, generally speaking ccRCC is a VHL-loss kidney cancer, and complete VHL inactivation is the quintessential first functional/genetic truncal event [7].

### The exemplary interconnected VHL/HIF/PBRM1/TSC/MTORC1 CBs in ccRCC

Among the three newly identified 3p21 TSGs in ccRCC, *PBRM1* is best studied in molecular mechanisms, mouse models, and human clinical outcomes [22, 28, 32, 42, 44–48]. Accordingly, we will further elaborate recent key research findings on PBRM1 and attempt to reconcile how and why PBRM1 loss in ccRCC impact the efficacy of select targeted therapies and immune checkpoint inhibitors.

#### PBRM1

The SWI/SNF are macromolecular protein complexes that utilize ATP to mobilize nucleosome, modulate chromatin structure, and thereby regulate central cellular, developmental and oncogenic processes [49–50]. They come with many flavors due to their interchangeable, dynamic compositions in nature [51]. Notably, mutations of individual SWI/SNF subunits are detected and exhibit preferential enrichment in ~20% human cancer of various types [52]. PBRM1 is the defining component of the PBAF complex and is most commonly mutated in ccRCC [53]. Remarkably, the *in vivo* tumor suppressor role of PBRM1 in ccRCC was confirmed and reported in 2017 by three independent laboratories using three different genetically engineered mouse models where combined losses of VHL and PBRM1 lead to multifocal ccRCC in mouse kidney, whereas individual losses did not [42, 54–55]. How PBRM1 loss might have contributed to the ccRCC pathogenesis will be discussed hereafter.

#### The hypoxia scenario: the normal physiology

**Figure 1:**
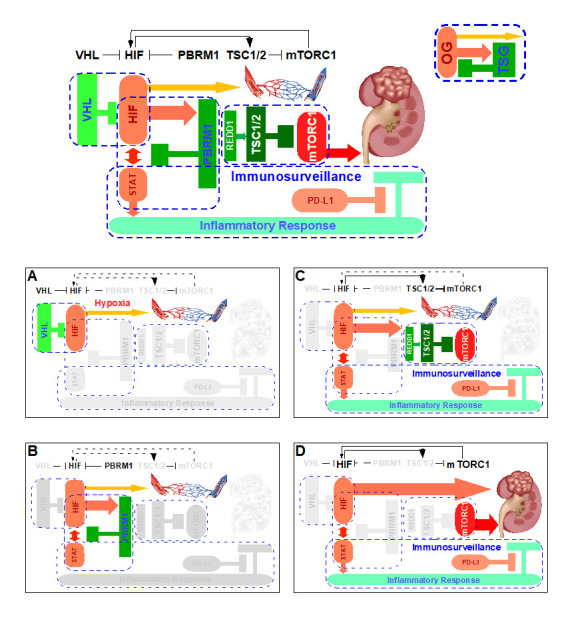
FIGURE 1: The interconnected OG-TSG CBs operate in renal epithelium to control signaling output and prevent tumorigenesis. **(A)** Depicts the physiological employment of the inherent VHL-HIF CB to gauge tissue response to low oxygen tension, and **(B-D)** examine how the sequential losses of a pre-determined set of “OG-TSG CBs” once the first CB is inactivated result in ccRCC.

Upon tissue injury, insufficient blood supply incurs, thereby resulting in low oxygen tension, which in turn stabilizes HIF-1 to act accordingly (**Fig. 1A**), i.e. inhibition of mitochondria oxidative phosphorylation and promotion of revascularization. Once the tissue repair is complete, normal oxygen tension is re-established, HIF-1 is prolyl hydroxylated which is recognized by VHL, ubiquitinated by VCB-Cullin 2 E3 ligase, and degraded by 26S proteasome, and tissue returns to a normal homeostatic state [34, 36].

#### The VHL loss scenario: inactivation of one CB

The complete pathologic loss of VHL due to chromosome 3p loss, mutations, and/or promoter methylation results in the aberrant abundance of HIF-1 protein, resulting in a hypoxia-like molecular response despite normal oxygen tension, i.e. pseudohypoxia. Under this scenario, the VHL loss inactivates the first ccRCC CB “VHL-HIF” (**Fig. 1B**) present in the renal cortex. *In vivo* mouse model studies demonstrated that the activation of pseudohypoxia program is evident when comparing the twelve week old normal appearing mouse renal cortex with kidney specific deletion of *Vhl* to that of wild-type control [42].

#### The VHL/PBRM1 loss scenario: inactivation of two CBs

The singular loss of VHL is insufficient in initiating ccRCC, which is recognized in both mouse and human VHL loss models [56]. The ensuing complete loss of PBRM1 inactivates the second CB “HIF-PBRM1-STAT” in the renal cortex (**Fig. 1C**), leading to the increased transcription output of HIF and STAT targets [42]. Of note, the singular loss of PBRM1 did not activate HIF or STAT targets [42]. It is known that HIF-1 and STAT3 cooperate to activate the expression of HIF-1 targets [57–58]. Consequently, the dysregulated interplay between HIF and STAT upon combined losses of VHL and PBRM1 creates a feed-forward amplification loop that maximizes downstream gene expression [42]. The role of PBRM1 in restricting HIF signaling output was independently reported using cell-based assays [59]. Under this scenario, the subsequent PBRM1 loss inactivates the second ccRCC CB “HIF-PBRM1-STAT”.

#### The VHL/PBRM1/TSC loss scenario: inactivation of three CBs

Despite the fact that HIF/STAT signaling overdrive was evident in the twelve week-old *Vhl-/-;Pbrm1-/-* mouse kidney, the long latency (ten months) and the incomplete penetrance (~50%) to the ultimate development of multifocal ccRCC in this model suggested that additional OG-TSG CBs could be at play [42]. To this end, gene expression and immunohistochemical analyses comparing twelve month-old ccRCC tumors to twelve week-old renal cortices of *Vhl-/-;Pbrm1-/-* mice detected hyperactive mTORC1 signaling in tumors in addition to the demonstrated pre-existing activation of HIF/STAT and suppression of mitochondrial pathways [42]. Hence, the prevention of aberrant activation of mTORC1 pathway probably constitutes the third ccRCC CB. Of note, mTORC1 serves as the central nutrition state integrator of the cell and its main control is conferred by the TSG TSC1/2 complex [60]. Remarkably, loss-of-function mutations in TSC1 or TSC2 (~10%), and gain-of-function mutations in MTOR (~6%) are common in ccRCC and correlated with rapalog response in therapeutic outlier studies; and *Tsc1* and *Tsc2* expression levels are down-regulated in *Vhl-/-;Pbrm1-/-* mouse ccRCC tumors [17, 22, 42, 61–63]. Accordingly, the third CB in place to prevent ccRCC pathogenesis after losses of VHL and PBRM1 is “TSC-mTORC1” (**Fig. 1D**). Of note, the inactivation of the “PTEN/PI3K” CB which functions upstream of the “TSC-mTORC1” CB is observed in 7% of ccRCC [22]. Mechanistically, the inactivation of the first “VHL-HIF” and the second “HIF-PBRM1-STAT” CBs and the resulting HIF output overdrive apparently activates *Redd1*, a known HIF1 target and an activator of TSC2 [42, 64], which positions “TSC-mTORC1' as the preferred third CB after the losses of VHL and PBRM1 [56].

#### A hypothetical immunosurveillance CB in ccRCC

As STATs are key transcription factors in cancer inflammation and immunity [65], the activation of STAT pathway due to the combined loss of VHL and PBRM1 could render the resulting tumors prone to immune regulation [42]. Recent approvals of single agent Nivolumab (anti-PD-1 antibody) as second line and the combination of Ipilimumab (anti-CTLA-4 antibody) and Nivolumab as first line treatment options for ccRCC have dramatically altered the therapeutic landscape of metastatic kidney cancer [66–67]. Intriguingly, a recent paper identified PBRM1 loss as a potential genomic biomarker for the treatment response to these immune checkpoint inhibitors [68–69] and others suggested otherwise [70–71], which needs further validation facing the daunting intratumor heterogeneity of ccRCC. Nevertheless, these mechanistic, mouse, and human ccRCC studies support a working hypothesis in which the disarmed “STAT-PD-1” immunosurveillance CB can be reactivated through biological means such as anti-PD-1/L1 antibodies for therapeutic exploitation (**Fig. 1D**). This might be one of the important rationales of why immunotherapy has activity in ccRCC, a tumor generally associated with low tumor mutation burden and a lack of microsatellite instability.

## FUTURE DIRECTION

### Integrated applied pathology to exploit functional pathology in advancing precision cancer therapeutics

The holy grail of contemporary cancer research is to be able to predict not only how an individual patient may benefit from currently available front-line therapies, but also how an individual tumor's molecular identity could potentially inform resistance mechanisms and thereby help implement a novel, tailored combination therapeutic strategy to greatly improve clinical outcome. One of the most challenging issues concerning metastatic ccRCC care is the known conspicuous intra-tumor and inter-tumor heterogeneity, which probably contributes to clinical outcomes. Nevertheless, like all individual human subjects develop from the same two copies of genome, all cancer cells evolve from the same set of genetic materials carried in the host cell. Hence, we hypothesize that intrinsic programming principles are in place to guide embryonic development, maintain tissue homeostasis, and restrict tumorigenesis [7, 14]. To visualize this third-generation hypothesis, we propose a “OG-TSG CB Constrained Braided Cancer River” model by integrating our first-generation “Braided River Model” and second-generation “CB Model” to further expound on this carcinogenic principle inspired from studying ccRCC, which might be applicable to additional cancer types.

#### The OG-TSG CB constrained braided cancer river model

**Figure 2:**
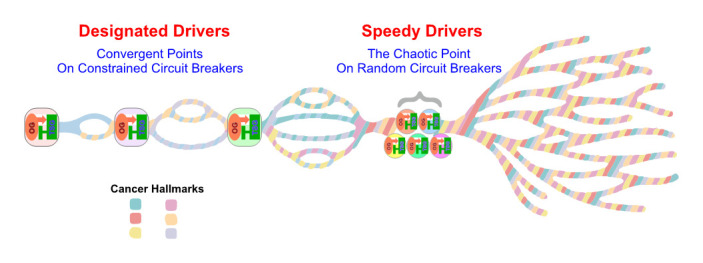
FIGURE 2. The diagram depicts the “OG-TSG CB Constrained Braided Cancer River” model to explain the non-randomness of cancer evolution and devise personalized cancer treatment strategy.

Despite conspicuous tumor heterogeneity, long-term clinical benefits on single agent targeted therapy are not uncommonly observed with metastatic ccRCC patients, implicating underlying cancer evolutionary constraints that force nonrandom sequences of parallel gene/pathway/function/phenotype convergences (**Fig. 2**). Indeed, our multi-region genomics study performed on ccRCC therapeutic outliers treated with single agent mTORC1 inhibitors rendered invaluable insights concerning this hypothesis [62]. We first proposed a braided cancer river model to help depict individual cancer evolution and advise therapeutic options.[7] The “Braided River” model highlights parallel and convergent events occurring throughout individual ccRCC tumorigenesis. It illustrates the stepwise, ordered accumulation of different driver mutations in kidney cancer development to acquire cancer hallmarks. A late chaotic evolutionary time point was incorporated to explain the limited effectiveness of combined targeted therapies in highly aggressive cancers, when “speedy” driver mutations quickly accumulate to enable extreme subclonal evolution. With the new concept of interconnected OG-TSG CBs, we propose a “CB-Constrained Braided Cancer River” model in which inherent context-dependent OG-TSG CBs are positioned at the gene/pathway/function/phenotype convergent points (**Fig. 2**). In other words, CBs function like dams to limit flow and prevent flooding. This model stipulates that each individual cancer river initiates with a truncal driver event, and once that occurs the ensuing branching events could be predicted. Accordingly, restoring a pre-determined, preferred set of CBs at once could offer effective measures and guide trial designs.

## Abbreviations:

CB: – circuit breaker;
ccRCC: – clear cell RCC;
HIF: – hypoxia inducible factor;
OG: – oncogene;
mccRCC: – metastatic ccRCC;
RCC: – renal cell carcinoma;
TSG: – tumor suppressor gene;
VHL: – Von-Hippel Lindau.
